# Supplementary material for: Causal interplay between lactose intolerance and gut microbiota: a combined bidirectional Mendelian randomization and in vivo validation study
Source: Front Nutr. 2026 Jun 1;13:1803337. doi: 10.3389/fnut.2026.1803337 (PMC13265576; doi:10.3389/fnut.2026.1803337)
Supplement: Supplementary file 1 [file Data_Sheet_1.zip › supplementary materials/Forward/leave one out cross validation/ebi-a-GCST90027521.finngen_R12_E4_LACTONAS.pdf]

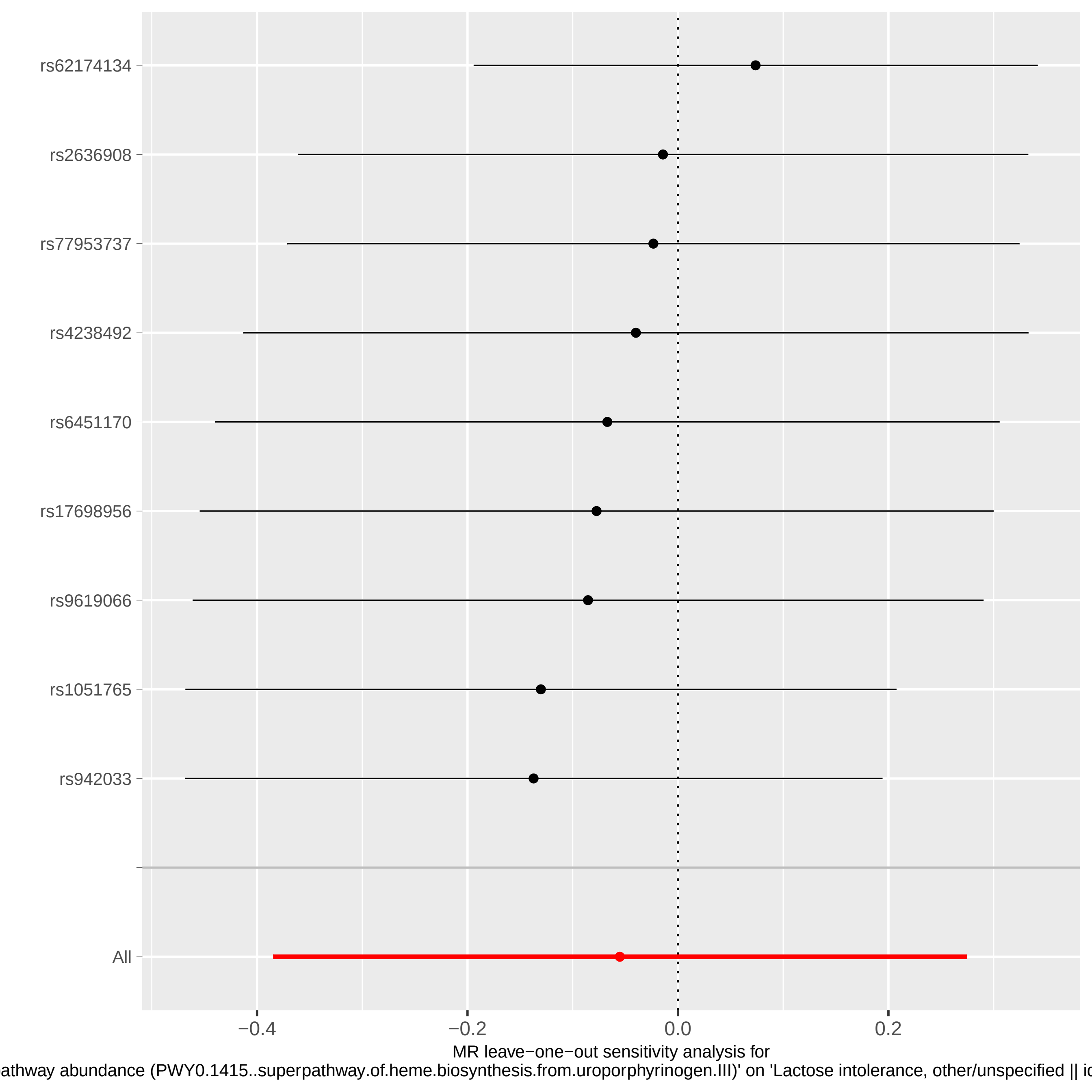

rs62174134
rs2636908
rs77953737
rs4238492
rs6451170
rs17698956
rs9619066
rs1051765
rs942033
All
−0.4
−0.2
0.0
0.2
MR leave−one−out sensitivity analysis for
athway abundance (PWY0.1415..superpathway.of.heme.biosynthesis.from.uroporphyrinogen.III)' on 'Lactose intolerance, other/unspecified || id
